# Antigen-Specific Mammary Inflammation Depends on the Production of IL-17A and IFN-γ by Bovine CD4+ T Lymphocytes

**DOI:** 10.1371/journal.pone.0137755

**Published:** 2015-09-16

**Authors:** Pascal Rainard, Patricia Cunha, Marion Ledresseur, Christophe Staub, Jean-Luc Touzé, Florent Kempf, Florence B. Gilbert, Gilles Foucras

**Affiliations:** 1 UMR1282, Infectiologie et Santé Publique, INRA, Nouzilly, France; 2 UE1297, Unité Expérimentale de Physiologie Animale, UEPAO, INRA, Nouzilly, France; 3 Université François Rabelais de Tours, Tours, France; 4 UMR0085, Physiologie de la Reproduction et du Comportement, INRA, Nouzilly, France; 5 INP, ENVT, Université de Toulouse, Toulouse, France; 6 UMR1225, Interactions Hôte Agents Pathogènes, INRA, Toulouse, France; Auburn University, UNITED STATES

## Abstract

Intramammary infusion of the antigen used to sensitize cows by the systemic route induces a local inflammation associated with neutrophil recruitment. We hypothesize that this form of delayed type hypersensitivity, which may occur naturally during infections or could be induced intentionally by vaccination, can impact the outcome of mammary gland infections. We immunized cows with ovalbumin to identify immunological correlates of antigen-specific mammary inflammation. Intraluminal injection of ovalbumin induced a mastitis characterized by a prompt tissue reaction (increase in teat wall thickness) and an intense influx of leukocytes into milk of 10 responder cows out of 14 immunized animals. The magnitude of the local inflammatory reaction, assessed through milk leukocytosis, correlated with antibody titers, skin thickness test, and production of IL-17A and IFN-γ in a whole-blood antigen stimulation assay (WBA). The production of these two cytokines significantly correlated with the magnitude of the milk leukocytosis following the ovalbumin intramammary challenge. The IL-17A and IFN-γ production in the WBA was dependent on the presence of CD4+ cells in blood samples. In vitro stimulation of peripheral blood lymphocytes with ovalbumin followed by stimulation with PMA/ionomycin allowed the identification by flow cytometry of CD4+ T cells producing either IL-17A, IFN-γ, or both cytokines. The results indicate that the antigen-specific WBA, and specifically IL-17A and IFN-γ production by circulating CD4+ cells, can be used as a predictor of mammary hypersensitivity to protein antigens. This prompts further studies aiming at determining how Th17 and/or Th1 lymphocytes modulate the immune response of the mammary gland to infection.

## Introduction

Infection of the MG of dairy ruminants by pyogenic bacteria such as streptococci, staphylococci and coliforms is a common disease and a major economic problem for milk producers [1]. The immune system is generally able to cope with mammary gland infections, but clinical mastitis and subclinical chronic infections are frequent [2]. Mastitis manifests itself by milk leukocytosis, and the early recruitment of neutrophils after bacterial entry into the lumen of the gland is of prime importance for the outcome of infection [3]. Although the innate immune system is efficient at triggering an inflammatory response in the mammary gland [4, 5], hypersensitivity has long been suspected to contribute to the inflammatory response of the mammary gland to common mastitis-causing bacteria [6]. It has been shown that sensitization of the mammary gland to staphylococcal antigens augments the initial recruitment of neutrophils into mammary glands inoculated with *Staphylococcus aureus* and increases the bactericidal activity of the recruited neutrophils [7–9]. Little is known of the mechanism of this sensitization, but it has been shown that the mammary gland antigen-specific response (mASR) can be transferred to naïve guinea pigs by adoptive transfer of lymphocytes but not by immune serum, leading the authors to conclude that the mASR was a local manifestation of a general state of lymphocyte-dependent hypersensitivity [10, 11].

Interest in the mASR has been fueled by its implications in terms of mastitis severity and after-effects. The possibility of its induction by vaccination in order to check bacterial proliferation in the mammary gland has been envisaged [3]. Its mechanism remains unknown, but hypotheses can be made based on recent advances in cell-mediated immunity. Neutrophilic inflammation can be initiated or reinforced by antigen-reactive T lymphocytes of the Th17 helper T cell lineage and its signature cytokine IL-17A [12, 13]. The Th17 lineage is particularly implicated in the defense of epithelial barriers against extracellular bacteria, through the recruitment of neutrophils at infection sites and its effects on epithelial cells [14]. A few circumstantial evidence substantiate that Th17 cells might contribute to the immune defense of the bovine mammary gland, and we suspect that Th17 cells contribute to mASR [15–18]. It has been shown that mammary epithelial cells are important contributors to the sensing of bacteria and the initiation of inflammation through innate immunity mechanisms [19–21], and that these cells are able to respond to the cytokines IL-17A and IL-17F in synergy with bacterial agonists of the innate immune system [22, 23]. The rationale for doing this study was to characterize mammary hypersensitivity, with a particular emphasis on the T cells that could be implicated in mASR. One objective was to find immunological correlates of mASR by looking for correlations between immune assays such as two-fold skin thickness test, whole blood antigen re-stimulation, or antibody response and the intensity of milk leukocytosis.

We sensitized cows with the model antigen ovalbumin and triggered inflammation by infusing the antigen into the mammary gland through the teat canal, as this has been proved to induce an intense inflammatory reaction [18, 24]. As we suspect that mASR involves Th17 lymphocytes, we empirically tried to favor a Th17-oriented immune response by fortifying the oil adjuvant with curdlan, a bacterial cell wall component reported to bias the immune response toward the Th17 axis [25, 26]. As this was not substantiated in the bovine, we scheduled a control group immunized with the oily adjuvant but without curdlan. By monitoring the response to immunization and the inflammatory reaction to the intramammary challenge, we found that milk leukocytosis correlated strongly with antigen-specific production of IL-17A and IFN-γ upon re-stimulation of the whole blood of the sensitized cows, and that this cytokine production was dependent on CD4+ T lymphocytes. Shortly after immunization, we found in peripheral blood CD4+ T cells producing IL-17A and IFN-γ. These results are in keeping with our hypothesis that Th17 cells contribute to the neutrophilic inflammation that develops in the mammary gland of sensitized cows, and prompt further study to delineate the nature of the T lymphocytes that are responsible for this phenomenon.

## Materials and Methods

### Ethics statement

All animal experiments were performed with approval from the Comité d’éthique pour l’expérimentation animale du Val de Loire (CEEA VdL), protocol number 2012-10-12 V2. Animal studies were compliant with all applicable provisions established by the European directive 2010/63/UE.

### Animals and experimental scheme

A preliminary experiment involved two groups of 3 heifers that were immunized with 10 μg pyrogen-free ovalbumin (Calbiochem) with either Incomplete Freund adjuvant (IFA, Sigma) or Montanide^TM^ ISA 61 VG (Seppic, Puteaux, France) supplemented with 100 μg Curdlan AL (a beta-1,3-glucan from *Alcaligenes faecalis*; InvivoGen, Toulouse, France) as adjuvant. The cows were immunized by subcutaneous (IFA group) or intramuscular (Montanide^TM^ group) on 3 occasions 60 days apart. In addition 3 cows were used as unimmunized controls. The immune response was monitored by taking blood samples to measure the antibody response and the production of IL-17A and IFN-γ in the antigen-specific whole blood assay (WBA) (see below), and by carrying out a skin test 15 days after the third immunization. This pilot experiment aimed at choosing the adjuvant used for the main experiment.

For the main experiment, 14 cows in their first or second lactation from a Teaching and Research herd (LEGTA, Domaine d’Areines, Vendôme, France) were selected on the basis of low somatic cell counts in udder quarter milk (< 200 000 cells/ml milk), absence of intramammary infection by major pathogens (*Staphylococcus aureus*, *Escherichia coli* or streptococci), and the occurrence of at least three uninfected quarters (no detectable bacterial growth in 50 μL milk samples). Challenged quarters were not infected and shed less than 100 000 cells/ml milk at time of challenge.

**Fig 1:**
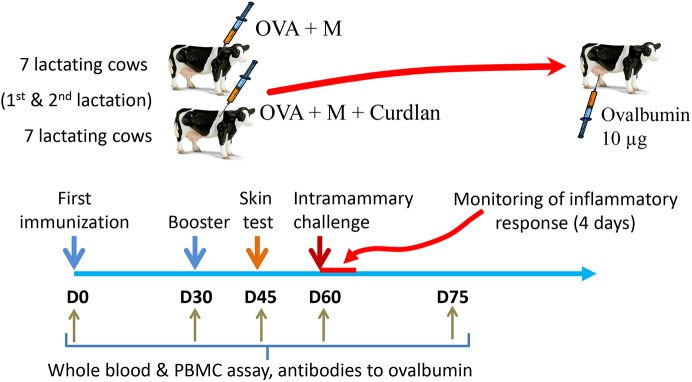
Experimental scheme. Two groups of 7 cows were immunized with ovalbumin (OVA) in two different adjuvant preparations. The immune response of the cows was monitored by taking blood samples on days 0, 30, 45, 60 and 75 days post-immunization. A skin test was performed 45 days post-immunization, and cows were challenged in one quarter with ovalbumin on day 60 post-immunization. The inflammatory response to the challenge was monitored for 4 days. M: Montanide ISA 61 VG.

Cows were allocated to two groups of 7 animals (Fig 1). The cows of one group received in the prescapular region 30 days apart 2 intramuscular injections of 50 μg (first injection) then 10 μg (booster injection) of pyrogen-free ovalbumin (Calbiochem) in 0.8 mL phosphate-buffered saline emulsified (water in oil emulsion) in 1.2 mL of Montanide^TM^ ISA 61 VG (Seppic) as adjuvant. The cows of the other group were immunized with ovalbumin by using the same regimen except that the antigenic preparations were supplemented with 100 μg Curdlan AL (InvivoGen).

The humoral immune response to immunization was monitored by measuring antibodies to ovalbumin in blood serum, and the cell-mediated immune response by carrying out a skin test 15 days after the booster immunization and by performing whole blood antigen stimulation assays at intervals (Fig 1). Cows were challenged with ovalbumin in the mammary gland 30 days after the booster immunization, and the local and systemic inflammatory response was monitored for 4 days.

### Intramammary antigen challenge

Cows were challenged by infusing ovalbumin into the lumen of one quarter through the teat canal. A solution of 10 μg/mL pyrogen-free ovalbumin (Calbiochem) was prepared in Hank’s buffered salt solution (HBSS) with 1 mg/mL pyrogen-free bovine serum albumin as described [18]. Immediately after the evening milking, 1 mL of the freshly made ovalbumin solution was infused through the teat canal by using a sterile smooth cannula fitted to a 1-mL disposable syringe whose plunger was vigorously pushed. Then the teat was massaged upward to ensure the diffusion of the ovalbumin dose.

### Examination of udders and milk samples

Following intramammary infusion of ovalbumin, clinical examination of the cows included visual assessment of udder swelling, sensitivity to palpation, and examination of the first streams of milk samples to look for flakes or discoloration. Body temperature was monitored with the Vel’Phone® sensor system (Medria Elevage, France). A Vell’Phone® thermometer was inserted into the vaginal cavity of every cow one week before challenge to measure body temperature at 30-min intervals. They were retrieved at the end of the follow-up period.

Aseptically taken foremilk samples were collected prior to challenge and 14, 48, 64, and 96 h post-infusion (hpi) with ovalbumin. Bacteriological analysis was performed throughout the experiments by plating 50 μL milk over sheep blood-esculin agar, and milk cell counts were measured with an automated cell counter (Fossomatic model 90; Foss Food Technology, Hillerod, Denmark) as described [27]. Proportions of neutrophils and mononuclear cells were determined by microscopic examination of cytospin slides prepared and stained with May-Grünwald-Giemsa as described [9]. Then the milk samples were centrifuged at 1,500 x *g* for 30 min at 4°C. Skimmed milk between the supernatant (fat layer) and the pellet (debris and cells) was harvested and stored in portions at -20°C until used to determine cytokine concentrations by ELISA.

### ELISAs for CXCL8, TNF-a, IFN-γ, IL-4, IL-10, IL-17A and IL-21

Enzyme-linked immunosorbent assays (ELISAs) for the chemokine CXCL8 and the cytokines TNF-α and IL-17A were performed as previously described [18, 28, 29]. IFN-γ and IL-4 concentrations were determined with commercially available bovine IFN-γ or IL-4 ELISA kit (MAbtech AB, Nacka Strand, Sweden). Concentrations of IL-10 were determined by using the matched antibody pair for bovine ELISA distributed by AbD Serotec (BioRad) and recombinant bovine IL-10 as standard (Kingfisher Biotech). Concentrations of IL-21 were determined by using the antibodies of the Do-It-Yourself ELISA and the recombinant bovine IL-21 commercialized by Kingfisher Biotech. The lower limits of detection for CXCL8, IFN-γ, TNF-α, IL-10, and IL-21 in milk by the ELISAs were 33, 10, 40, 15 and 200 pg/ml, respectively.

### Ultrasonographic examination

Ultrasonography were performed by the same operators using an Esaote Piemedical MyLab30 ultrasound device equipped with a linear LA332 probe and a volumetric BL433 probe (all materials from Hospimedi France, Valdampierre, France). Ultrasonography of individual teats was performed at 5 MHz immediately after milking by placing the probes along the teat skin, taking great care not to crush or flatten the teats. Data were acquired on the four quarters of each cow one week before challenge to select teats without morphological defects. Teats displaying cistern inflammation before challenge were excluded from the experiment. Ultrasonography measurements were carried out on the challenged and contralateral quarters just before challenge and at 16, 48, 96 and 168 hpi.

### Ovalbumin skin test

As an assessment of the cell-mediated immune response, the cutaneous delayed-type hypersensitivity to OVA was measured by using a skin test. Two weeks after the booster injection of OVA, all cows were given an intradermal injection of 10 μg OVA in pyrogen-free saline. Injections were administered in the right or left side of the neck using a 28 gauge needle. Prior to injections, the sites were shaved using an electric shaver and cleaned with 70% ethanol. Double skin-fold thickness measurements of each site were made using a spring-loaded caliper just before the injections and 24, 48 and 72 h later. Results were expressed as increase in skin thickness compared to pre-injection thickness.

### Antibody titers to OVA

**A**ntibodies to ovalbumin were measured in blood serum by indirect ELISA. Antibody titers were determined in the IgG1 and IgG2 subclasses. Microtiter plates (Nunc Immunoplate Maxisorp) were coated by overnight incubation with 2 μg/ml ovalbumin (100 μL/well) in phosphate-buffered saline (PBS), before saturation with a solution of 5 mg/mL fish gelatin (Sigma) in PBS (PBSG) for 1 h at 37°C. Following each incubation step, the plates were washed three times with PBS supplemented with 0.1% (v/v) Tween 20 (Sigma) with a plate washer (MW96/384, Beckman Coulter^TM^). Sera were diluted in PBSG 1/1000 for IgG_1_ determination or to 1/250 for IgG_2_ determination, taking into account a lower response in the IgG2 than in the IgG1 sub-isotype. These dilutions fell within the range of concentration determination of the standard curve obtained with a reference serum. Dilutions were distributed in the plate (100 μL/well) and incubated for 2 h at 37°C. Then the plates were incubated for 60 min at 37°C with a 1/10000 dilution of either sheep anti-bovine IgG_1_ or sheep anti-bovine IgG_2_ conjugated to horseradish peroxidase (AbD Serotec BioRad), and finally the reaction was revealed with 3,3’,5,5’-tetramethylbenzidine enzyme substrate (Uptima, Interchim, Montluçon, France). To determine antibody titers, a standard curve was established in each plate with a series of dilutions of the serum from a cow hyperimmunized with ovalbumin, rich in IgG2 antibodies to ovalbumin. This serum was arbitrarily given a titer of 1000 IgG_1_ units and 5000 IgG_2_ units. Concentrations of antibodies were calculated with the software provided with the ELISA reader manufacturer (Labsystems, Helsinki, Finland).

### Whole blood antigenic stimulation

Blood was collected from the caudal blood vessels into commercially available 10-mL evacuated tubes coated with lithium-heparin as anticoagulant (Venosafe™, Terumo® Europe). Samples were used within 4 h after collection. Stimulations were performed in triplicate by mixing in 96-well microplates (Falcon Microtest™, Becton Dickinson) 100 μL of blood with either 100 μL of culture medium (RPMI-1640 supplemented with 10% fetal bovine serum, 2 mM L-glutamine, 10 mM HEPES, penicillin-streptomycin, fungizone and 0.05 mM 2-mercaptoethanol) as negative control, 100 μL of pokeweed mitogen (2 μg/mL) as positive control, or 100 μL of ovalbumin solution (50 μg/mL). The culture was incubated at 37°C in a humidified atmosphere with 5% CO_2_ for 3 days unless specified otherwise (see Figure legends). Supernatant were then harvested and stored at -20°C in 96-well plastic storage plates (Greiner™ bio-one) until assayed for cytokine content.

### Isolation of peripheral blood mononuclear cells (PBMC)

PBMC were isolated from blood samples collected in 10-mL blood collection tubes coated with EDTA (Venosafe™, Terumo® Europe). The tubes were centrifuged at low speed (400 x *g*) for 10 min at 20°C and the upper layer of plasma containing platelets was discarded. The tubes were then centrifuged at 1000 x *g* for 10 min at 20°C, and the buffy coat and the upper part of the red pellet were diluted 1/5 with DPBS and transferred onto a Percoll cushion (Percoll mixed with DPBS to obtain a density of 1.077) in a 15 mL centrifuge tube. After centrifugation (400 x *g*, 15 min, 20°C, brake off), the upper white cell layer was washed twice in DPBS supplemented with 10% FBS, and the cells were finally adjusted to 6 x 10^6^ cells/2 mL in culture medium.

PBMC were depleted of CD4+ T cell by using MACS® beads according to the manufacturer's instructions (Miltenyi Biotech, Bergish Gladbach, Germany). Briefly, cells (2 x 10^7^) were incubated with a mouse anti-bovine CD4 (Table 1). After washing, the cells were incubated for 20 min under mild agitation with anti-mouse IgG MACS Microbeads in MiniMACS buffer (PBS, 2 mM EDTA, 0.5% bovine serum albumin). The cells were washed and labeled with an anti-mouse IgG1 antibody conjugated to PE (Jackson ImmunoResearch Lab.). After washing, a portion of the cells was put aside for assessment of the % CD4+ cells and WBA, and the bulk was passed over a MACS® (MS) separation column mounted on an OctoMACS® Separator to isolate the CD4+ T cells. Cytometric analysis (see below) indicated that the fall-through cell fraction contained on average 0.5% CD4+ cells, compared to more than 10% in the PBMC preparations before depletion. WBA was performed on depleted and unfractionated PBMC.

**Table 1:** Antibodies used for flow cytometry analysis.

Antigen	Primary antibody	Provider	Dilution	Secondary antibody	Provider	Dilution
CD4	AlexaFluor647-conjugated mouse MAb anti-bovine (clone CC8)	AbD Serotec, Oxford, UK	1/100			
CD4	Mouse Mab anti-bovine (clone CC30)	AbD Serotec, Oxford, UK	1/1000	perCPCy5.5 Goat anti-mouse IgG	Biolegend, London UK	1/100
IL-17A	Rabbit polyclonal anti-bovine	Kingfisher, Biotech Saint Paul, USA	1/200	RPE conjugated Donkey anti rabbit IgG(H+L)	Interchim, Jackson ImmunoResearch	1/100
IL-17A	PE-conjugated mouse monoclonal anti-human (eBio64DEC17)*	eBioscience, Hatfield UK	1/20			

*this antibody has been reported to cross-react with sheep IL-17A [30]

### Cytometric analysis

When appropriate, PBMC were stimulated in 96-well microplates as for the WBA, by using 100 μL of PBMC suspension and 100 μL of OVA (20 μg/mL) per well, with pokeweed mitogen (2 μg/mL) and only culture medium as positive and negative controls, respectively. The plates were incubated at 37°C in a humidified atmosphere with 5% CO_2_ for 3 days, and the supernatant was harvested and stored at -20°C until assayed for cytokine content. The cells were washed and adjusted to 10^6^ cells/mL in culture medium and incubated at rest at 37°C for 2 days. Then, the cells were stimulated for 5 h with 50 ng/mL phorbol 12-myristate 1-acetate (PMA) and 500 ng/mL ionomycin (Sigma), in the presence of Brefeldin A (Sigma) for the last 3 h. When appropriate, cells were stained with a live/dead cell fixable stain kit (fixable viability Dye eFluor 660, eBioscience), according to the manufacturer’s instructions. Cells were surface stained with anti-CD4 Ab, fixed, permeabilized, and stained with anti-IL-17A and anti-IFN-γ (Bio-Rad, AbD Serotec) Abs using the Cytofix/Cytoperm kit (BD Pharmingen). Data were acquired on a FACScalibur cytometer using Cellquest software (BD Biosciences) and analyzed with FlowJo software (Tree Star, Ashland, OR, USA). Gates were set according to appropriate isotype/control staining.

### Statistical analysis of data

Statistical analyses were performed with the Prism software (GraphPad, version 5.0). A probability level of < 0.05 was considered significant. Comparisons of paired samples were done with the Friedman’s test followed by Bonferroni post-test comparison when appropriate. Correlation analyses were done with the Spearman’s rank test. A principal component analysis (PCA) was performed with the ADE4 software [31] to summarize the variance observed in each of the following variable: decrease in teat cistern volume, IFN-γ in milk 14 hpi, IgG1 titer D45, IgG2 titer D45, IgM D45, IL-17A in milk 48 hpi, Peak SCC, Skin test D45 24h, Temperature 24 hpi, WBA IFN-γ D45, WBA IL-17A D45. A permutation test was used to estimate the correct number of components to retain (10 000 permutations of measurements in each variable) [32]. This was done using the testdim option of R-package ADE4 [31]. The randtest function was next used to test if individuals clustered according to the immunization treatment (i.e. M or M+C). To this end, the between-groups inertia percentage was compared to the distribution estimated by a Monte Carlo permutation procedure (1000 permutations).

## Results

### Preliminary experiment

A pilot experiment was designed to help us determine if we could replace IFA by a better tolerated adjuvant. The IFA has been reported to be suitable to induce mASR in ruminants as efficiently as complete Freund adjuvant [24], but there was no published data regarding the use of the Montanide^TM^ ISA 61 VG. We supplemented the Montanide^TM^ adjuvant with Curdlan as this bacterial wall extract containing a beta-1,3-glucan has been reported to favor antigen-specific neutrophilic inflammation in a mouse model [25]. We compared the immune response of heifers immunized with ovalbumin emulsified in either IFA or Montanide + curdlan (3 animals/group). We found no difference in the OVA-specific IgG1 antibody response, but a trend for a stronger response in the IgG2 isotype (S1A & S1B Fig). Both preparations induced a reaction in the skinfold test with a maximal response 24 h post-injection, whereas there was little if any reaction on the part of the control cows (S1C Fig). There was a trend for a more consistent or stronger response in IL-17A and IFN-γ production in the antigen-specific WBA (S1D Fig). We opted for the Montanide adjuvant because this adjuvant is better tolerated than is IFA at site of injection and because it is authorized for use in ruminants. As we did not know whether curdlan contributed to the observed responses, we set up the main experiment to make possible the assessment of its effect by immunizing two groups of cows, one with and the other without curdlan.

### Mammary response to OVA challenge

In the main experiment, the intramammary infusion of 10 μg of pyrogen-free OVA into one quarter induced local signs of inflammation in 10 of the 14 immunized cows (responders). Inflammation was detected in the responsive cows at 24 h up to 72 hours post-infusion (hpi) with redness, moderate swelling and tenderness. Signs of mastitis had disappeared by 96 hpi. Small clots became obvious in milk from these quarters at 24 hpi, and greatest changes (flakes) were usually seen at 48–72 hpi. Milk appearance was back to normal by 96 hpi. Quarters adjacent to the infused quarters were visually normal. During the experimentation, none of the cows contracted an infection in the challenged or control quarters.

**Fig 2:**
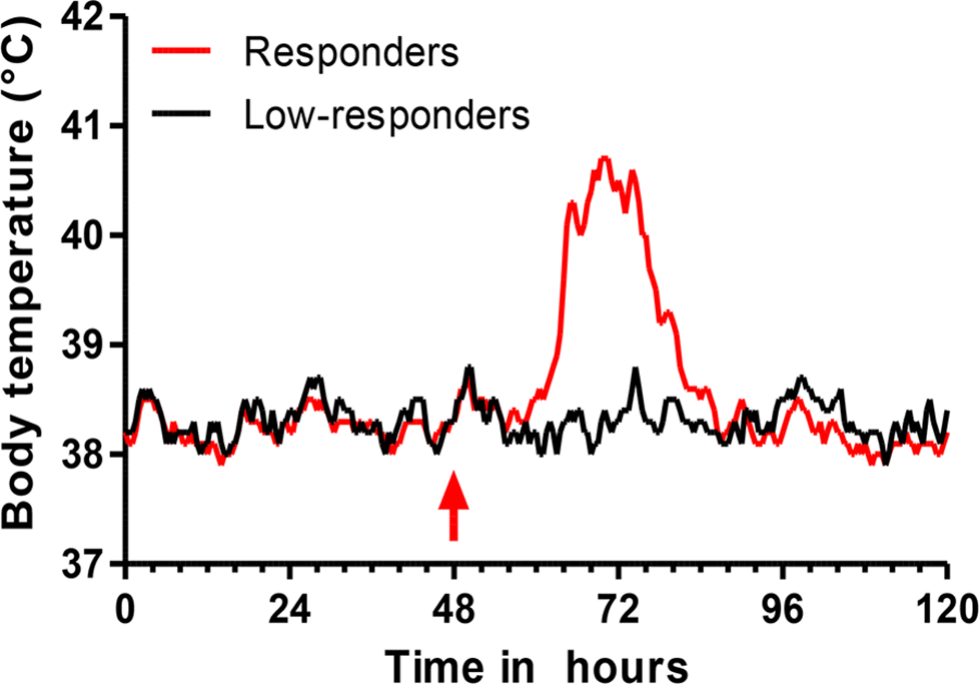
Monitoring of the bodily temperature with an intravaginal sensor system. Median values of body temperature of high responder and low-responder cows before and after the intramammary challenge with OVA at 48 h (red arrow). Minor ticks indicate 4-hour intervals.

All but one of the responder cows, but none of the low-responders, experienced a rise in temperature following the OVA challenge. On average, hyperthermia began 12 hpi, peaked around 40.5°C at 20–28 hpi, and ended by 36 hpi (Fig 2).

**Fig 3:**
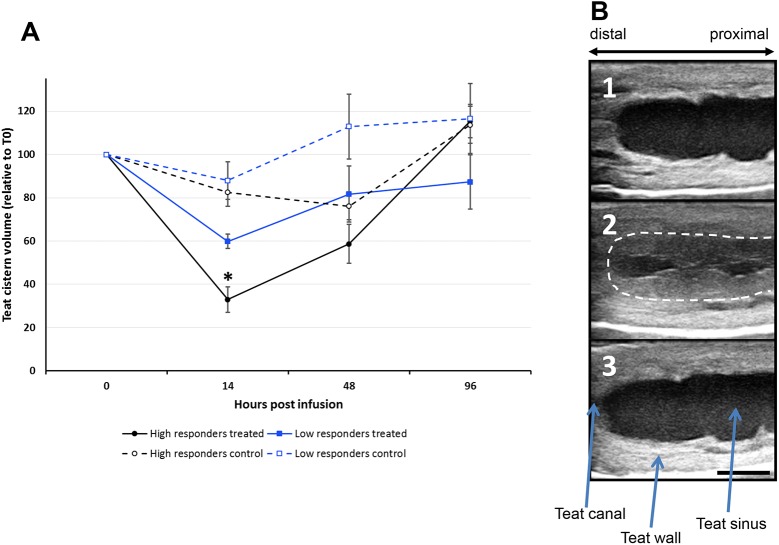
Ultrasonographic assessment of the teat cistern volume following ovalbumin infusion. **(**A) Graph representing the evolution of the teat cistern volume (mean values from 10 responders and 4 low responders, ± standard deviations) relative to T0 (before ovalbumin infusion) showing a significant reduction of the teat cistern volume 14 hours after ovallbumin infusion into teat cisterns of high-responder cows (*p<0.05); (B) Ultrasounds photographs representing an ovalbumin-infused teat before infusion; (1): the cistern is filled with liquid (milk) therefore weakly echogenic; (2) 14 hpi, there was a thickening and permeabilization of the wall of the teat (inflammation) reducing the cistern volume. The white dashed line represents the initial position of the cistern wall; (3) 96 hpi, the teat has regained its original appearance. The bar represents 1 cm.

All but one of the 10 responder cows and 1 of the 4 low-responder cows experienced a sharp decrease in teat cistern volume following the ovalbumin intramammary challenge (Fig 3A). This volume reduction resulted from the thickening of the teat wall (Fig 3B). There was a significant reduction of the volume of the teat cistern 14 hours after the ovalbumin treatment in high responders treated quartier compared to the volume of the same teat cistern before ovalbumin infusion (p<0.05) and compared to contralateral control quartier at the same time (p<0.05). The difference in volume reduction between challenged quarters of responders and low-responders was not significant. Teats recovered 48 h after ovalbumin treatment as high and low responders treated cistern volume retrieved 60% and 80% of their initial volume respectively. The Spearman’s correlation of teat cistern volume decrease with peak SCC was poor (R = 0.266), was significant with the IL-17A production (R = 0.566, p = 0.025) but not with IFN-γ production (R = 0.330, p > 0.05) in the WBA at D45, when one outlier was excluded.

### Milk leukocytosis and cytokine concentrations in response to intramammary challenge

**Fig 4:**
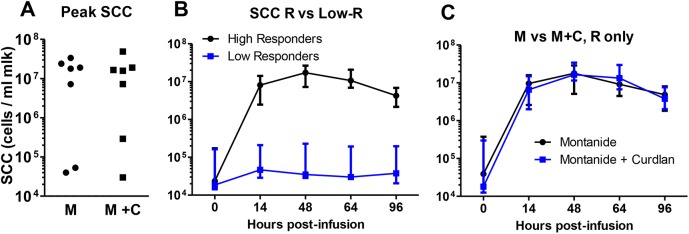
Concentrations of cells (SCC, somatic cell count) in milk after intramammary infusion of ovalbumin. (A) Peak SCC, i.e. highest cell concentrations reached at either 14 or 48 hours post-infusion in the infused quarter milk of cows of the Montanide (M) and the Montanide + Curdlan (M+C) groups. (B) Time-course of the influx of cells into infused quarter milk of the 14 cows distinguishing high-responder and low-responder cows. (C) Time course of cell influx into the infused quarter milk of only high-responder cows of each immunization group.

The intramammary infusion of OVA triggered an intense influx of leucocytes into the milk of 10 out of 14 challenged cows (Fig 4A). Milk cell concentrations were already high at the first post-infusion time (14 hpi) and peaked at 48 hpi (Fig 4B). There were two non-responder cows in each of the immunized groups (Fig 4A), and the kinetics of the milk leukocytosis was not influenced by the addition of curdlan to the Montanide^TM^ adjuvant (Fig 4C). The examination of stained milk cytospins showed that most of the recruited leukocytes were neutrophils, with a proportion of mononuclear cells that increased with time from 5–10% to 25–35% when the inflammation subsided (results not shown), as previously described [18].

**Fig 5:**
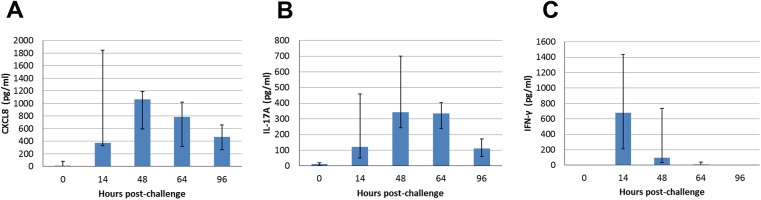
Concentrations of cytokines in milk samples of the 10 responder cows. Time-course of concentration variation (median and interquartiles) of CXCL8 (A), IL-17A (B) and IFN- γ (C) in the milk of quarters infused with ovalbumin at 0 hpi.

There was no significant difference in milk concentrations of CXCL8, IFN-γ or IL-17A related to the use of curdlan (results not shown). For the analysis of cytokine concentrations in milk of the challenged quarters, all cows were considered as belonging to the same immunized group. The chemokine CXCL8, a chemoattractant for neutrophils and a marker of inflammation, was detected in the milk of the responder cows at the first sampling (14 hpi), with peak concentrations at 48 hpi (Fig 5A). The cytokine IL-17A was found in the milk of all the high-responder cows from 14 hpi, concentration peaked at 48–64 hpi and was still noticeable at 96 hpi (Fig 5B). Concentration of IFN-γ increased in the milk of all the responders but one (the cow that did not develop hyperthermia) with a peak at 14 hpi followed by a brisk decline (Fig 5C). Low-responders had no detectable levels of CXCL8, IL-17A and IFN-γ in milk. The cytokines TNF-α and IL-10 were not detected in milk at any time.

### Antibody responses to ovalbumin

**Fig 6:**
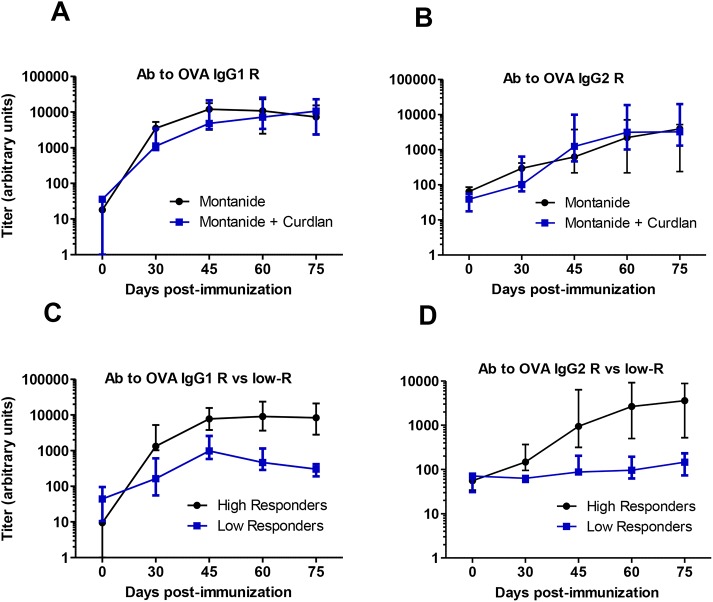
Time-course of the humoral response following immunization with ovalbumin at days 0 and 30. (A & B) Variations of antibody titers (IgG1 and IgG2) in serum samples of the responder cows (R) of the two immunization groups in the IgG1 and IgG2 subclasses. (C & D) Variation of antibody titers (IgG1 and IgG2) in serum samples of responder and low-responder cows (low-R).

The quantification of serum antibodies to OVA by ELISA revealed that all of the responder cows developed high titers in the IgG1 and IgG2 sub-isotypes, and that the addition of curdlan to the adjuvant had no effect (Fig 6A and 6B). Considering the absence of curdlan effect on the inflammatory (milk leukocytosis and cytokine concentrations) and antibody responses, the two immunization groups were merged for the other analyses of the immune response. Low-responder cows developed low titers of IgG_1_ antibodies and did not show increases in IgG_2_ antibodies to OVA (Fig 6C and 6D). For responder cows, titer increases occurred earlier and were higher in the IgG_1_ than in the IgG_2_ isotype (Fig 6C and 6D). The correlations (Spearman’s rank test) between the SCC values at the peak of milk leukocytosis and antibody titers at 45 days post-immunization averaged 0.8 and were highly significant (Table 2). IgG titers correlated also highly with the production of IL-17A and IFN-γ in the WBA carried out on day 45 post-immunization (Table 2).

**Table 2:** Correlations (Spearman’s rank test) between IgG_1_ or IgG_2_ and Peak SCC and the OVA-specific WBA.

	IgG_1_ D45	IgG_2_ D45
Peak SCC	R = 0.833 p = 0.0002	R = 0.807 p = 0.0005
WBA IL-17A D45	R = 0.833 p = 0.0002	R = 0.749 p = 0.002
WBA IFN-γ D45	R = 0.930 p < 0.0001	R = 0.798 p = 0.0006

Antibody titers and WBA performed on samples taken on day 45 after the first immunization (D45).

### Ovalbumin skin test

**Fig 7:**
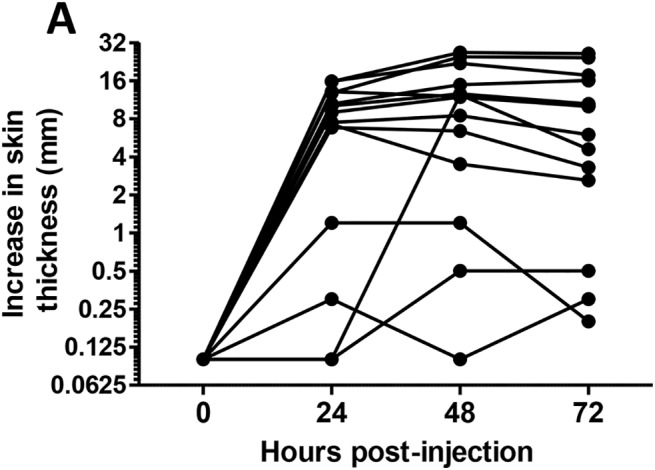
Kinetics of increases in skin thickness following intradermal inoculation with ovalbumin of the 14 immunized cows. (A) Increases in skinfold thickness calculated by subtracting the thickness value measured before inoculation from the values measured after injection. (B) Spearman correlation between Peak SCC and skin test values measured at 24 or 48 h post-inoculation, considering either the 14 immunized cows (All) or only the 10 responders (R). ns: not significant (p > 0.05).

The preliminary experiment allowed us to establish that the intradermal injection of 10 μg of pyrogen-free OVA to unimmunized cows did not induce a noticeable swelling at the site of injection (S1C Fig). By contrast, all the immunized responder cows reacted to the intradermal injection of OVA with a large increase in skin thickness (Fig 7). The increase was already at its most at 24 hpi and plateaued until 72 hpi. In 3 of the low-responder cows, the increase in skin thickness was equal to or less than 1 mm, except one animal which showed a protracted response at 48 and 72 hpi (Fig 7). The Spearman correlation between the skin test values and the peak SCC was high, and higher for values at 24 hpi (0.752, p = 0.002) than for values at 48 hpi (0.626, p = 0.017). When only the responder cows were considered for analysis, the correlation was much lower and not significant. These results suggest that the skin test was able to distinguish responders from low-responders, but was not able to rank the responder cows.

### Antigen-specific WBA

**Fig 8:**
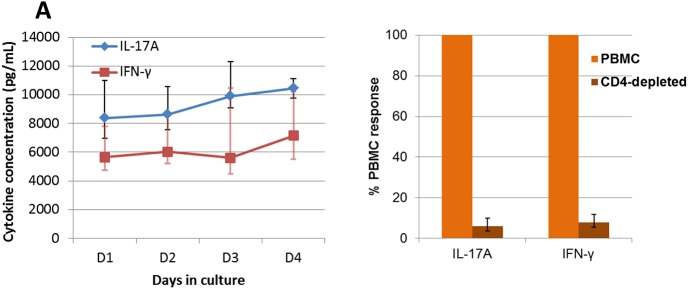
Antigen-specific whole blood assay. **(**A) Time-course of IL-17A and IFN-γ production by whole blood cultured with OVA. The blood of the 10 responder cows (taken 45 days after the first immunization) was cultured in the presence of OVA (10 μg/mL) for 1 to 4 days in 96-well microplates. For each cow triplicate wells were used for each incubation time. Results are median values and quartiles (Q1; Q3). (B) The effect of magnetic depletion of CD4+ cells on the production of IL-17A and IFN-γ in the antigen-specific whole blood assay. Results obtained by stimulating blood samples from responder cows 15 days after the booster immunization are expressed as the percentage of the production by CD4+-depleted PBMC relative to cytokine production by un processed PBMC (100%). The viability of cells was not altered by the magnetic cell separation.

Preliminary experiments indicated that the anticoagulant of choice for the WBA was lithium-heparin and for PBMC isolation EDTA. In the negative control wells receiving only culture medium, cytokine concentrations were either undetectable or very low, and in positive controls with pokeweed mitogen, cytokine concentrations were in the 7 to 20 ng/mL range for both IL-17A and IFN-γ (results not shown). Concentrations of IL-17A and IFN-γ were monitored during the culture of whole blood with OVA for up to 4 days, which showed that cytokine production was already high after one day, and that concentrations tended to increase slightly over the follow-up period (Fig 8A). It is generally considered that IL-17A and IFN-γ are produced mainly by T lymphocytes. To identify the source of cytokine production in response to OVA stimulation, PBMC of 4 responder cows were depleted of CD4+ cells. The production of IL-17A and IFN-γ was only a few percent (0.5 to 17%) of the cytokine production by the unfractionated PBMC (Fig 8B). This result established that the production of IL-17A and IFN-γ was CD4+ cell dependent, indicating that the two cytokines were mainly produced by CD4+ T lymphocytes.

**Fig 9:**
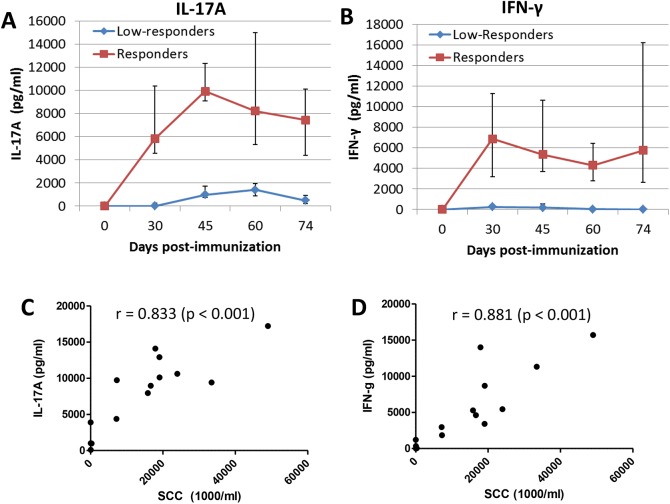
Time-course of the IL-17A and IFN-γ production in the antigen-specific whole blood assay following immunization and correlation with Peak SCC. Concentrations of IL-17A (A) or IFN-γ (B) after 3 days of culture with ovalbumin of blood samples taken before and after immunization at days 0 and 30 (median values and interquartiles) distinguishing the antigen-specific responses of responders and low-responder cows to the intramammary antigenic challenge. (B and C) Correlations (Spearman’s rank test) between peak SCC and IL-17A concentrations or IFN-γ concentrations yielded by the whole blood assay performed 45 days after the first immunization.

The production of IL-17A and IFN-γ in the OVA-specific WBA was monitored over the period spanning the immunization and challenge periods. Prior to immunization, none of the cows reacted to OVA. With the blood samples of high-responder cows, stimulation with OVA induced concentrations of both IL-17A and IFN-γ that were already high at the time of the booster immunization, which was followed by a further increase for IL-17A only (Fig 9A and 9B). There was no obvious recall effect of the intramammary OVA challenge at day 60 post-immunization (Fig 9A and 9B). By contrast, OVA-stimulation of low-responder cow blood samples induced only a low production of IL-17A and no significant production of IFN-γ (Fig 9A and 9B). Concentrations of IL-17A and IFN-γ calculated with data from the 14 cows over the observation period were only moderately correlated (R = 0.60, p = 0.0001).

The correlations (Spearman’s rank test) between the magnitude of mammary inflammation (Peak SCC) and the concentrations of IL-17A or IFN-γ (in the OVA-specific WBA) suggested a few considerations (Table 3). In keeping with the contrasted WBA result yielded by responders versus low-responders, the correlations between cytokine production in the WBA performed 45 days after the first OVA immunization and the antigen-specific mammary inflammation assessed through the peak SCC were high for the two cytokines (Fig 9C and 9D). Correlations obtained by using data (IL-17A and IFN-γ production at day 45 and Peak SCC) from responders only were high and significant, suggesting that the OVA-specific WBA was able to predict the magnitude of the inflammatory mammary response of the responder cows. Besides, the correlations between Peak SCC and the OVA-specific WBA were weaker at 60 days than at 45 days post-immunization. This suggests that the predictive value of the OVA-specific WBA is at its best only for some time after immunization, during a time window that will need to be determined. The existence of a time window is supported by the lack of correlation between Peak SCC and WBA performed at 60 days post-immunization when only responder cows were considered.

**Table 3:** Correlations (Spearman’s rank test) between Peak SCC and the OVA-specific WBA.

OVA-specific WBA	Spearman correlation with Peak SCC
WBA IL-17A D45 All cows	0.833 (p < 0.001)
WBA IL-17A D45 Responders	0.648 (p < 0.025)
WBA IL-17A D60 All cows	0.560 (p< 0.025)
WBA IL-17A D60 Responders	-0.200 ns
WBA IFN-γ D45 All cows	0.881 (p < 0.001)
WBA IFN-γ D45 Responders	0.782 (p < 0.01)
WBA IFN-γ D60 All cows	0.604 (p < 0.025)
WBA IFN-γ D60 Responders	0.030 ns

Data based on either the 14 cows under experiment (All cows) or the 10 responders (Responders). The highest SCC values were correlated with the concentrations of IL-17A or IFN-γ yielded by the OVA-specific WBA performed 45 (D45) or 60 (D60) days after the first immunization.

The correlations between the Peak SCC and the skin test were higher at the 24 h than at the 48 h reading (Table 4). This was also true for the correlations between the skin test and the OVA-specific WBA (Table 4). Correlations were higher with the IL-17A WBA performed at 45 days post-immunization than with those performed at 60 days (Table 4 and S2 Fig). Production of IFN-γ in the WBA performed at 45 and 60 days post-immunization was correlated with the skin test read at 24 h (Table 4). There was no correlation between production of IL-4 in the OVA-specific WBA and the skin test (Table 4).

**Table 4:** Correlations (Spearman’s rank test) between skin thickness, Peak SCC and OVA-specific WBA

	Skin test at 24 h	Skin test at 48 h
Peak SCC	0.752 (p = 0.002)	0.626 (p = 0.017)
WBA IL-17A D45	0.827 (p = 0.0002)	0.675 (p = 0.008)
WBA IL-17A D60	0.557 (p = 0.039)	0.477 (p = 0.085)
WBA IFN-γ D45	0.695 (p = 0.006)	0.516 (p = 0.059)
WBA IFN-γ D60	0.708 (p = 0.005)	0.618 (p = 0.019)
WBA IL-4 D45	-0.144 (p = 0.624)	-0.149 (p = 0.611)

Data based on the 14 cows under experiment. The skin tests values read 24 h or 48 h after ovalbumin intradermal inoculation were correlated with the concentrations of IL-17A or IFN-γ yielded by the OVA-specific WBA performed 45 (D45) or 60 (D60) days after the first immunization.

We also investigated the production of a type 2 immune response by measuring IL-4 secretion in the WBA at day 45 post-immunization. Low concentrations of IL-4 were found in supernatant cultures upon OVA stimulation (mean value 185 pg/mL), slightly more than the concentrations found in the control unstimulated cultures (57 pg/mL). The correlation between IL-4 concentrations (OVA cultures) and Peak SCC was low and not significant (r = -0.180, p = 0.54).

**Fig 10:**
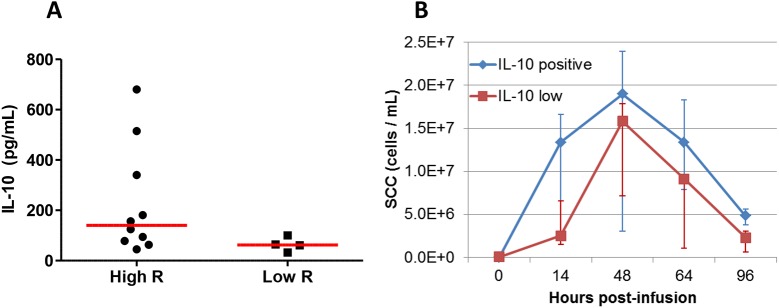
Production of IL-10 in the OVA-specific WBA did not correlate with mASR. (A) IL-10 concentrations in the WBA performed at 45 days on the 10 responders (High R) and the 4 non responders (Low R). (B) Comparison of the milk leukocytosis of the 5 highest producers of IL-10 with the 5 lowest IL-10 producers among the 10 high-responder cows. Results are medians and interquartiles (Q1; Q3). Medians are significantly different at 14 hpi (Mann and Whitney, p = 0.028).

We wondered whether the lack of immune response of the low responder cows could be related to the induction of OVA-specific IL-10-producing cells. Contrary to this hypothesis, OVA-specific WBA performed at 45 days post-immunization revealed that low-responders displayed the lowest production of IL-10 (Fig 10A). Among the ten high-responder cows, the five that produced the highest concentrations of IL-10 in the OVA-specific WBA tended to recruit cells in milk earlier with a significant difference at 14 hpi (p = 0.028) but with similar peak concentrations to the other five responders (p > 0.05) (Fig 10B).

**Fig 11:**
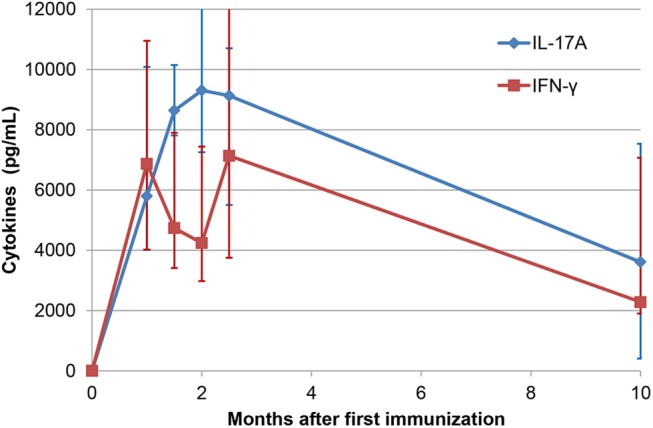
Persistence of reactivity to ovalbumin. Concentrations of IL-17A and IFN-γ yielded by the whole blood assay performed at different times after immunization with ovalbumin. Results are the median values (and interquartiles) from the 8 responder cows still available 10 months post-immunization.

Persistence of the OVA-specific response was evaluated by measuring the production of IL-17A and IFN-γ in the WBA of eight responder cows still in the herd 10 months after the first immunization. Concentrations of IL-17A and IFN-γ halved, showing that circulating antigen-reactive cells were still present 8.5 months after the last immunization (Fig 11).

### Cytometric analysis of blood lymphocytes

To characterize OVA-specific CD4 T cells in the blood of immunized cows, we developed the procedure that allowed us to label IL-17A-producing bovine T lymphocytes. For intracellular labeling of IL-17A, we used two antibodies (Table 1) that both enabled a clear distinction between labeled and non-labeled cells (S3 Fig). Nevertheless, the polyclonal antibody to bovine IL-17A (Kingfisher Biotech) labeled on average twice as many cells as did the monoclonal eBio64DEC17 (eBioscience).

**Fig 12:**
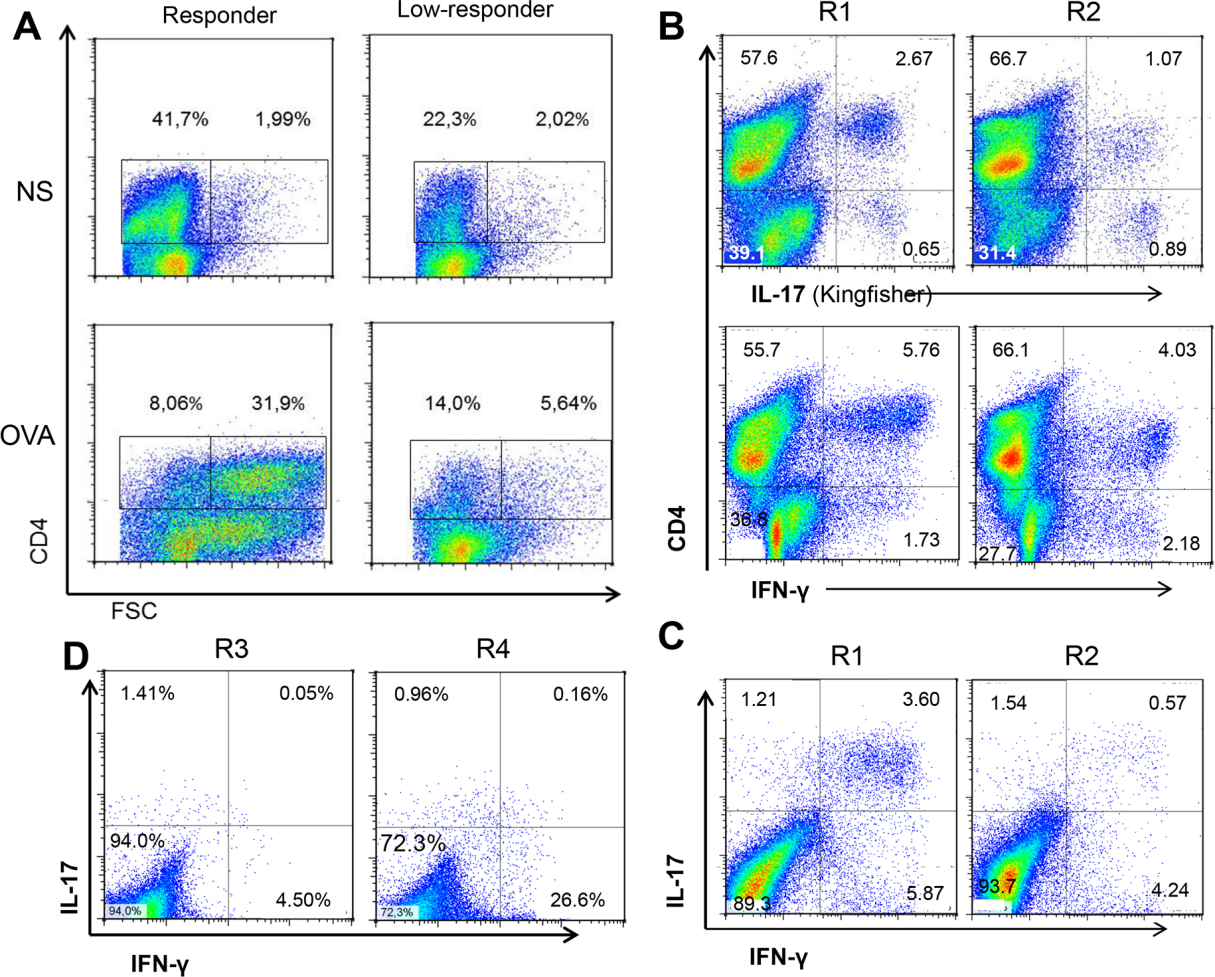
Intracellular expression of IL-17A and IFN-γ by CD4+ T lymphocytes. PBMC were isolated one month after ovalbumin booster injection, stimulated in vitro with ovalbumin for 3 days, rested for 2 days and finally stimulated with PMA/ionomycin for 5 h with Brefeldin A for the last 3 hours. Cells were labeled for surface CD4 and intracellular IL-17A and IFN-γ. The numbers in the plots indicate the percentages of labeled cells in comparison to the isotype control. (A) Production of viable CD4+ T lymphoblasts after culture of PBMC with (OVA) or without (NS) ovalbumin. Left panels depict PBMC from a responder cow, right panels PBMC from a low-responder. (B) PBMC from two responder cows (R1 & R2) were labeled for surface CD4 and intracellular IL-17A or IFN-γ, showing CD4+ and CD4- IL-17A- and IFN-γ-producing cells. (C) labeling of CD4+ cells with anti-IL-17A and anti-IFN-γ antibodies, showing single-producing and double-producing cells. D) Double labeling of CD4+ cells from two low-responders (R3 and R4). Percentages of labeled cells are indicated in the quadrants. Results are from a representative experiment.

Since IL-17A and IFN-γ production in the antigen-specific WBA were dependent on the presence of CD4+ cells, we sought for IL-17A and IFN-γ producing T lymphocytes in the blood of responder cows. Blood samples were taken from two responders about one month post-immunization, a time when the WBA yielded positive results for these two cytokines. PBMC stimulated for 3 days with ovalbumin, rested for two days, and stimulated with PMA/ionomycin with addition of Brefeldin A were stained for CD4 and intracellular IL-17A or IFN-γ. Under these conditions, CD4+ blast cells (high FSC) appeared in the ovalbumin-stimulated cultures of responder cows, but not in ovalbumin cultures of low-responders (Fig 12A). Intracellular labeling of PBMC from two responder cows revealed the presence of IL-17A or IFN-γ single positive cells (Fig 12B) and also some double-producing cells (Fig 12C). In addition low responders displayed considerable lower responses of double positive stained lymphocytes (0.05–0.16%) than did high responders (0.57–3.60%) (Fig 12C and 12D).

### Principal component analysis

**Fig 13:**
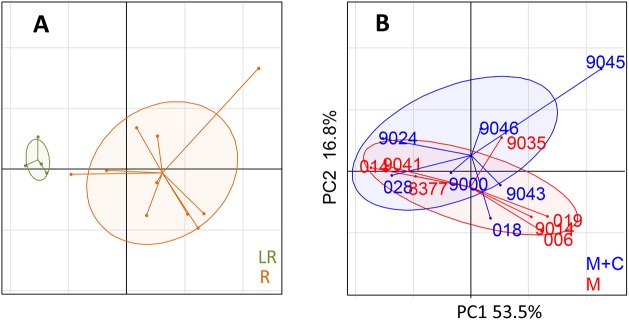
Principal component analysis of the inflammatory and immune parameters variability measured from the 14 immunized cows. The two components retained explained respectively 53.5% (p < 0.001) a,d 16.8% (p = 0.16) of the total inertia. (A) The ellipses representing the LR (Low-responders) and R (Responders) groups are clearly separated (p = 0.004). (B) The 1.5 standard deviation-inertia ellipses of the Montanide (red ellipse and cow numbers) and the Montanide + curdlan (blue ellipse and cow numbers) overlap extensively. The between-group test was not significant (p = 0.58).

To get an overall view of the relationship of the different inflammatory and immune parameters measured and found the most pertinent in individual analysis, we carried out a principal component analysis based on the peak SCC, OVA-specific antibody titers, skin test, WBA, body temperature and teat cistern volume, from the 14 cows under experiment. Clearly, the 4 low-responders were set apart from the 10 responders, and among the responders there was no clear-cut difference regarding the use of curdlan in the immunogenic preparation (Fig 13A). This analysis thus confirmed that curdlan did not modify the immune response to ovalbumin under our experimental conditions (Fig 13B).

## Discussion

This study was devised to investigate the hypothetical role of Th17 cells in the immune response of the mammary gland to infection by mastitis-causing bacteria. A possible manifestation of the antigen-specific Th17 contribution to mammary gland response is mASR, because IL-17A and Th17 cells are implicated in neutrophilic inflammation and can be induced by vaccination [25, 33]. The main objective of this experiment was to find immune correlates of mASR, with a particular emphasis on Th17 cells. We found that the production of IL-17A and IFN-γ by circulating CD4+ lymphocytes of immunized cows correlated with the intensity of milk leukocytosis. In this study, the antigen-specific WBA was a predictor of mASR, enabling the discrimination of responder and low-responder cows and the intensity of the neutrophilic inflammation in the ovalbumin model of mammary hypersensitivity. Delayed-type hypersensitivity has long been suspected to contribute to the immune response of the mammary gland to mastitis-causing pathogens, but its underlying mechanisms are still poorly understood and its consequences in terms of disease severity and outcome remain undetermined. Filling this gap would have implications in the understanding of the pathogeny of mastitis and in devising new therapeutic or preventive strategies.

### Development of the ovalbumin-specific WBA

To monitor the cell-mediated immune response to immunization with ovalbumin, we developed an antigen-specific whole blood stimulation assay. This assay is based on the same principle as the WBA IFN-γ release assays which is widely used to assess the cell-mediated immune response to immunization with BCG or bacterial components, or to infection by *Mycobacterium tuberculosis* or *M*. *avium subsp*. *paratuberculosis* [34–37]. To this assay we added the measurement of IL-17A production. We checked that IL-17A and IFN-γ production in the blood of responder cows was dependent on the presence of CD4+ T cells (Fig 8B). It has previously been reported that the OVA-specific in vitro responses of in vivo primed bovine PBMC was dependent on the presence of CD4+ T cells [38]. In this study we found a significant but moderate (R = 0.6) correlation between IL-17A and IFN-γ production following immunization with ovalbumin. Responder cows were still producing IL-17A and IFN-γ upon ovalbumin re-stimulation 10 months after the booster immunization, suggesting that circulation of memory cells is relatively long-lasting. Yet, we did not re-challenge these animals intramammarily, and thus cannot confirm that mASR is long-lasting.

A major finding of this study was that the production of IL-17A and IFN-γ by circulating CD4+ T cells of immunized cows correlated with mASR intensity, and circulating leukocytes of low-responder cows released very little if any IL-17A or IFN-γ. This suggests that the ovalbumin-specific WBA can be used as a predictor of the response to immunization with ovalbumin with regard to mASR. The absence of IL-4 release and the production of IFN-γ and IL-17A suggest that mASR is related to a Th1 and/or Th17-like immune response. Consequently the question arises of the nature of the cells that reacted to ovalbumin by producing IL-17A or IFN-γ, beyond the necessary presence of CD4+ cells.

We obtained some information on the cells at the origin of the antigen-specific production of IL-17A and IFN-γ in the whole blood assay and on the lymphocytes responsible for the mASR phenomenon, as the necessary reagents became available during the course of the study. We present the first evidence of bovine CD4+ T cells producing either IL-17A or IL-17A and IFN-γ (Fig 12). In vitro studies of blood-derived T cells showed that circulating T cells have the ability to produce either IL-17A or IFN-γ exclusively, or together, defining double-producing cells. These cells that produce both IL-17A and IFN-γ are suspected to be more pathogenic than IL-17A single producers because they induced stronger inflammatory reactions but may also be more efficient at combating infections [39]. Multifunctional T cells that secrete high amount of several cytokines are considered optimized for effector function and more efficient at controlling infections [40].

We hypothesize that Th17 cells are at the origin of the mASR phenomenon. The results of the present study are in line with this hypothesis, but they do not allow us to decide whether Th17 cells, Th1 cells, or if Th1/17 cells are necessary and sufficient to trigger an antigen-specific neutrophilic inflammation in the mammary gland. We found a high correlation between Peak SCC after antigen challenge and the antigen-specific WBA production of IL-17A and IFN-γ by CD4+ cells, thus supposedly between mASR and the presence of circulating Th17 and Th1 lymphocytes. As the mASR develops in a few hours after intramammary infusion of an antigen which on its own is non-pyrogenic, the cells responsible for the inflammation setting off are likely to be antigen-reactive cells already present in the mammary tissue when the antigen is captured from the lumen of the gland. These antigen-reactive cells are either re-circulating memory cells that can be detected by the antigen-specific WBA, or non-circulating tissue resident memory cells that elude the assay [41, 42]. Intriguingly, the correlation between WBA and peak SCC was higher at D45 than at D60, suggesting that the cells producing IL-17A and/or IFN-γ circulate for some time after immunization but begin to disappear from the circulation a few weeks after immunization. Possibly the D45 WBA is a good correlate of mASR because it is concomitant with the seeding of the mammary tissue by the effector T lymphocytes responsible for the mASR.

The seeding of mammary tissue with T lymphocytes may be reflected by the T cells that can be retrieved from mammary gland secretions, as a result of egress into the gland lumen. We endeavored to identify IL-17A and IFN-γ-producing lymphocytes in milk during mASR, but our attempts to analyze milk lymphocytes by cytometry were fruitless due to co-aggregation with activated neutrophils. This suggests an intense stimulation of neutrophils and a highly activated status. Activation of neutrophils and associated increased microbicidal activity may be useful to control infection. Indeed, the increase in the bactericidal activity of the neutrophils recruited in milk at the beginning of the mASR has been reported [9]. The cytokines IL-17A and IFN-γ were found in milk as soon as milk leukocytosis developed (Fig 5). This may impact the course of infection, as IFN-γ can activate bovine phagocytes [43], and IL-17A has been shown to augment the response of mammary epithelial cells to stimuli of bacterial origin [22].

### Absence of effect of curdlan on mASR, WBA and Ab titers

According to the Th17 hypothesis, we evaluated the effect of supplementation of the antigen preparation with curdlan on the immune response to ovalbumin, as this β-glucan has been reported to favor the generation of Th17 cells, although there is no consensus on this activity [25, 26, 44]. We did not find any effect of curdlan supplementation on the proportion of low-responders, on antibody titers, skin test or WBA. The overall absence of effect was confirmed by the principal component analysis of the data (Fig 13). This lack of activity may have resulted from a poor activity of curdlan on bovine dendritic cells. This result does not mean that β-glucans are devoid of interest for Th17 orientation in the bovine. Investigations on the effect of β-glucans from different sources on bovine dendritic cells would be necessary to select a potentially better promoter of Th17-type immune response.

### Low-responders

In this study we had confirmation that about a third of the cows immunized with ovalbumin did not respond, as in our previous study, although Montanide^TM^ adjuvant has been used rather than IFA [18]. The low-responders were detected by the antigen-specific WBA and by the skin test measured at 24 h post-injection, and they did not develop antibodies in the IgG_2_ subclass (Fig 6). Among the possible causes for this absence of response there is the Major Histocompatibility Class II repertoire, or tolerance to ovalbumin. Cows are supposed to be naïve to ovalbumin, and our results are in keeping with this hypothesis, since very low IgG titers and very little if any production of IFN-γ and IL-17A in the antigen-specific WBA were found before immunization. The existence of OVA-specific regulatory cells cannot be excluded, but we did not find a correlation between the production of IL-10 in the antigen-specific WBA and the mASR (Fig 10). Uncovering the reasons behind such a high proportion of low-responders was out of the scope of this study, but would deserve attention in case a similar proportion would be induced by a vaccine immunogen.

### Correlations of skin test and antibody responses with mASR

Besides the antigen-specific WBA, mASR (peak SCC) correlated with the skin test and the antibody titers. The skinfold thickness test serves as a classic evaluation of delayed-type hypersensitivity. In our study, the correlations between the Peak SCC and the skin test were higher at the 24 h than at the 48 h reading (Table 3): this may suggest that the 24 h reading was more related to the production of IL-17A than the later reading, and as this also applied to the Peak SCC, that the mechanism underlying mASR was more related to the early phase of the skin reaction. In the skin test when neutrophils are attracted, they migrate before mononuclear leukocytes, and this is probably related to the neutrophilic character of mASR. Skinfold thickness can result from distinct immune mechanisms, and thus is less specific than the IL-17A/IFN-γ production of the antigen-specific WBA, which for this reason is a better candidate for mASR correlate. Teat wall thickening may share some underlying mechanisms with the skin thickness reaction. The resulting teat cistern shrinking was an early event, showing that the epithelium and underlying tissue were equipped to detect and to react to the infused antigen. The teat has been shown to be reactive to infection and can be considered as a sentinel of the mammary gland [45, 46]. The correlation of the reduction in teat cistern volume with mASR intensity was nevertheless rather poor and not significant. Antibody titers also correlated with mASR and thus could be used as an indicator of this type of immune response. Nevertheless, antigen-specific WBA is probably a better correlate of mASR, because Ab are not likely to contribute directly to mASR, contrary to CD4+ T lymphocytes. Consequently, the WBA relation with mASR is probably more robust.

### Conclusion, interest and implications of the results

The magnitude of the reaction of the mammary gland to intramammary injection of small amounts of a soluble protein devoid of pyrogenic property on its own is intriguing. The mammary gland is not reputed to be an organ prone to allergy or hypersensitivity. Yet, like the skin, the mammary gland has been shown to be subject to antigen-specific acute inflammation. This supposes that the mammary gland is equipped with all the cells necessary to mount a swift and intense response to antigens. This capacity and the role of mASR in the response to infection are probably undervalued. We found that the antigen-specific production of IL-17A and IFN-γ by CD4+ T cells is a predictor of the antigen-specific immune response of the mammary gland. This finding is in keeping with the hypothesis that Th17 cells contribute to the neutrophilic inflammation that can be induced in the mammary gland by vaccination, and prompts further studies to delineate the nature of the T lymphocytes that are responsible for this phenomenon and to determine whether either IL-17A or IFN-γ or both are necessary for mASR. Our research findings provide a better understanding of antigen-specific immune responses in mammary glands and opens up the possibility to harness vaccination-induced mASR to defend against mammary gland infections.

## Supporting Information

S1 Fig Immune responses to ovalbumin obtained with either Incomplete Freund Adjuvant (IFA) or Montanide^TM^ ISA 61 G as adjuvants. Three heifers were immunized with ovalbumin in IFA (IFA 1, 2, 3) or Montanide^TM^ ISA 61 (Mont 1, 2, 3), on days 0, 30 and 60, and three cows were used as unimmunized controls (T 1, 2, 3). A & B) IgG1 and IgG2 titers were monitored by ELISA. C) Increases in skinfold thickness (mm) calculated by subtracting the thickness value measured before inoculation from the values measured after inoculation. D) Production of IFN-γ and IL-17A in the antigen-specific whole blood assay. Blood samples were taken 75 days after the first immunization, and the culture supernatant were collected after 3 days of stimulation.
(PDF)

S2 Fig Correlations (Spearman rank test) between the skin test performed at day 45 post-immunization and the OVA-specific WBA performed at days 45 or 60 post-immunization. (PDF)

S3 Fig Intracellular labeling with antibodies to IL-17A. PBMC were isolated from the blood of an immunized cow, restimulated in vitro with ovalbumin for 3 days or left unstimulated, rested for 2 days and finally stimulated with PMA/ionomycin for 5 h with Brefeldin A for the last 3 hours. Cells were then labeled to reveal intracellular IL-17A with either rabbit antiserum to bovine IL-17A (Kingfisher Biotech) followed by RPE-conjugated anti-rabbit antibody, or PE-conjugated mouse monoclonal antibody to human IL-17A (eBioscience). A) isotype control; B) unstimulated cells; C) ovalbumin-stimulated cells. Viable cells are shown.
(PDF)
