# Supplementary figures and images for: Antigen-Specific Mammary Inflammation Depends on the Production of IL-17A and IFN-γ by Bovine CD4+ T Lymphocytes
Source: PLoS One. 2015 Sep 16;10(9):e0137755. doi: 10.1371/journal.pone.0137755 (PMC4573518; doi:10.1371/journal.pone.0137755)

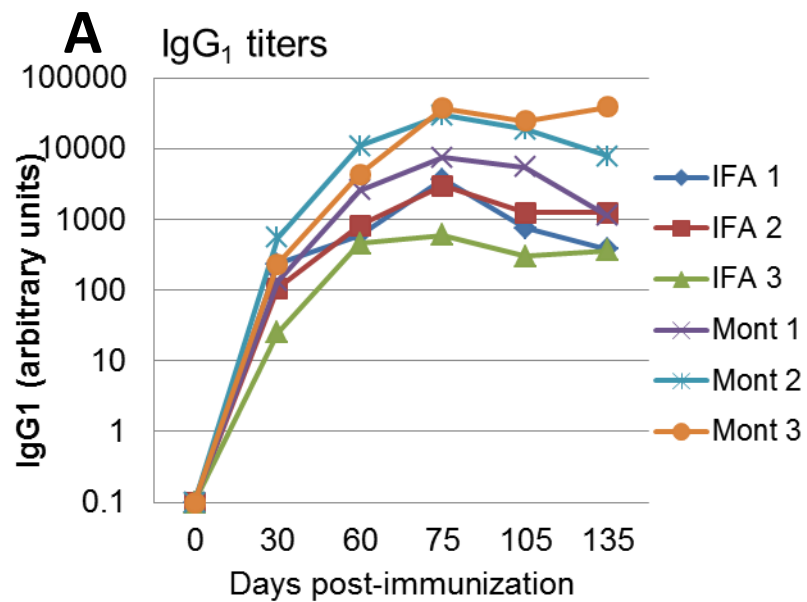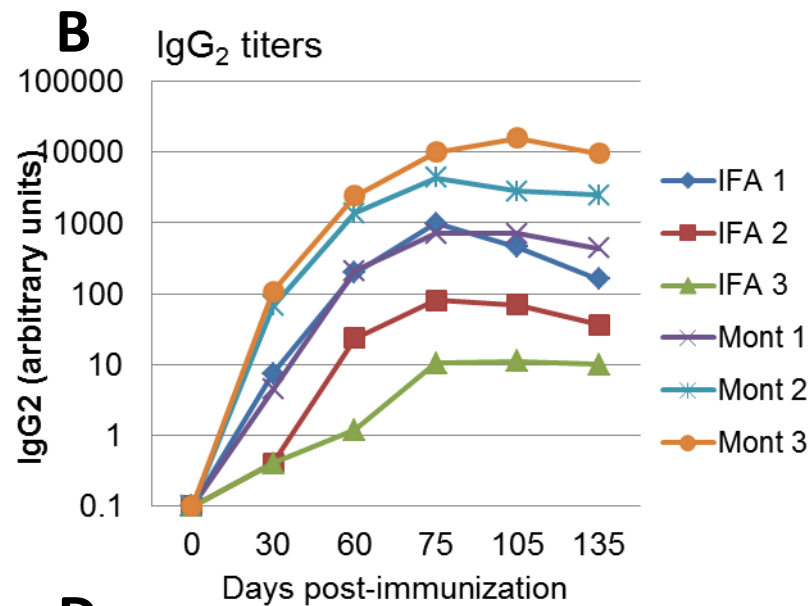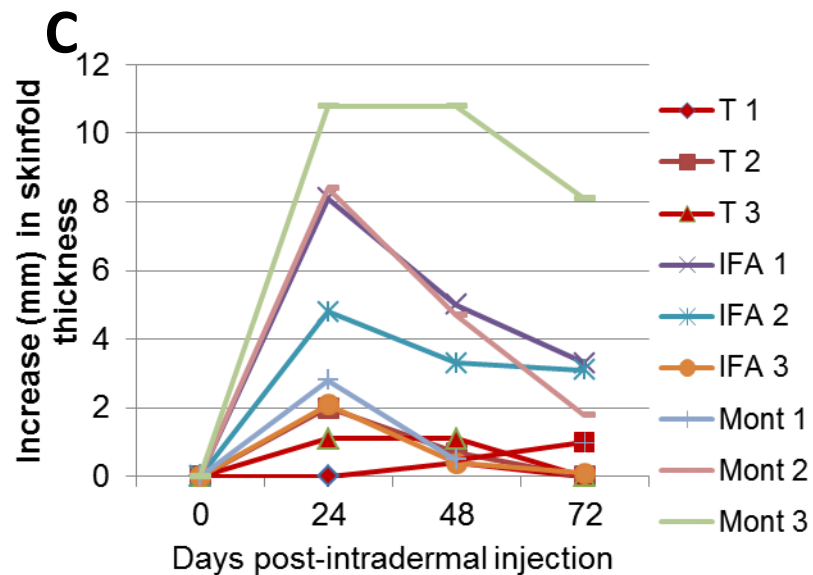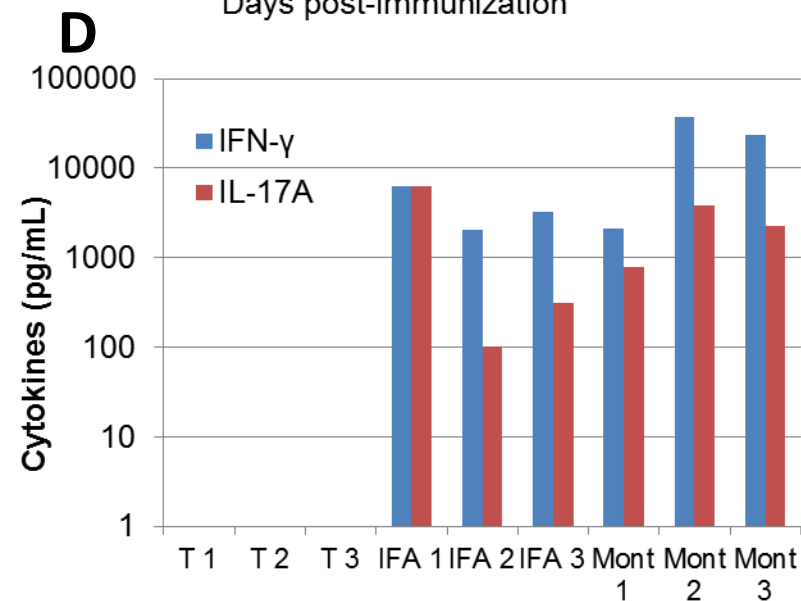

Supplement: S1 Fig — Three heifers were immunized with ovalbumin in IFA (IFA 1, 2, 3) or MontanideTM ISA 61 (Mont 1, 2, 3), on days 0, 30 and 60, and three cows were used as unimmunized controls (T 1, 2, 3). A & B) IgG1 and IgG2 titers were monitored by ELISA. C) Increases in skinfold thickness (mm) calculated by subtracting the thickness value measured before inoculation from the values measured after inoculation. D) Production of IFN-γ and IL-17A in the antigen-specific whole blood assay. Blood samples were taken 75 days after the first immunization, and the culture supernatant were collected after 3 days of stimulation. (PDF) [file pone.0137755.s001.pdf]
