# Supplementary material for: Antigen-Specific Mammary Inflammation Depends on the Production of IL-17A and IFN-γ by Bovine CD4+ T Lymphocytes
Source: PLoS One. 2015 Sep 16;10(9):e0137755. doi: 10.1371/journal.pone.0137755 (PMC4573518; doi:10.1371/journal.pone.0137755)

Correlation blood assay / skin test (Spearman)

	IL-17A	IFN-γ	IL-4
Skin test 24 h	0.827 (p < 0.01)	0.695 (p < 0.01)	0.14 p = 0.62
Skin test 48 h	0.675 (p < 0.01)	0.516 (p = 0.058)	

IL-17A

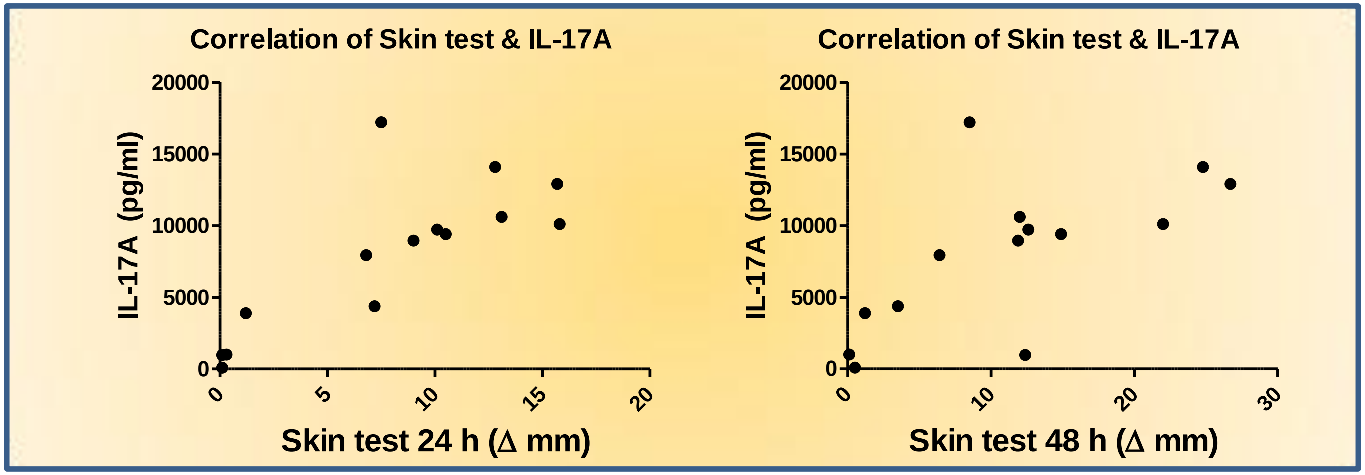

INF-γ

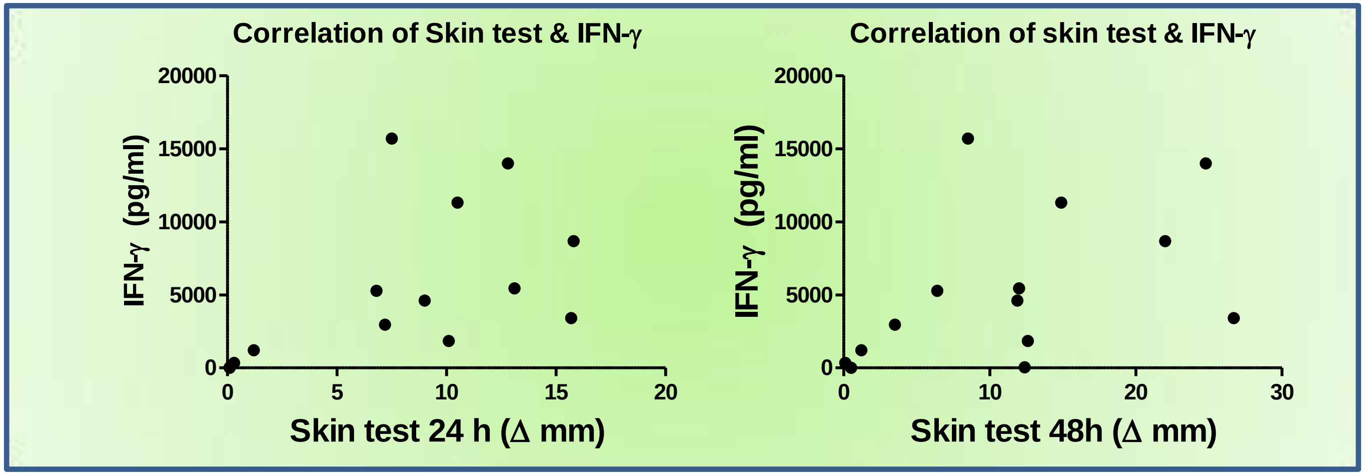

Supplement: S2 Fig — (PDF) [file pone.0137755.s002.pdf]
