# Supplementary material for: Antigen-Specific Mammary Inflammation Depends on the Production of IL-17A and IFN-γ by Bovine CD4+ T Lymphocytes
Source: PLoS One. 2015 Sep 16;10(9):e0137755. doi: 10.1371/journal.pone.0137755 (PMC4573518; doi:10.1371/journal.pone.0137755)
Supplement: S3 Fig — PBMC were isolated from the blood of an immunized cow, restimulated in vitro with ovalbumin for 3 days or left unstimulated, rested for 2 days and finally stimulated with PMA/ionomycin for 5 h with Brefeldin A for the last 3 hours. Cells were then labeled to reveal intracellular IL-17A with either rabbit antiserum to bovine IL-17A (Kingfisher Biotech) followed by RPE-conjugated anti-rabbit antibody, or PE-conjugated mouse monoclonal antibody to human IL-17A (eBioscience). A) isotype control; B) unstimulated cells; C) ovalbumin-stimulated cells. Viable cells are shown. (PDF) [file pone.0137755.s003.pdf]

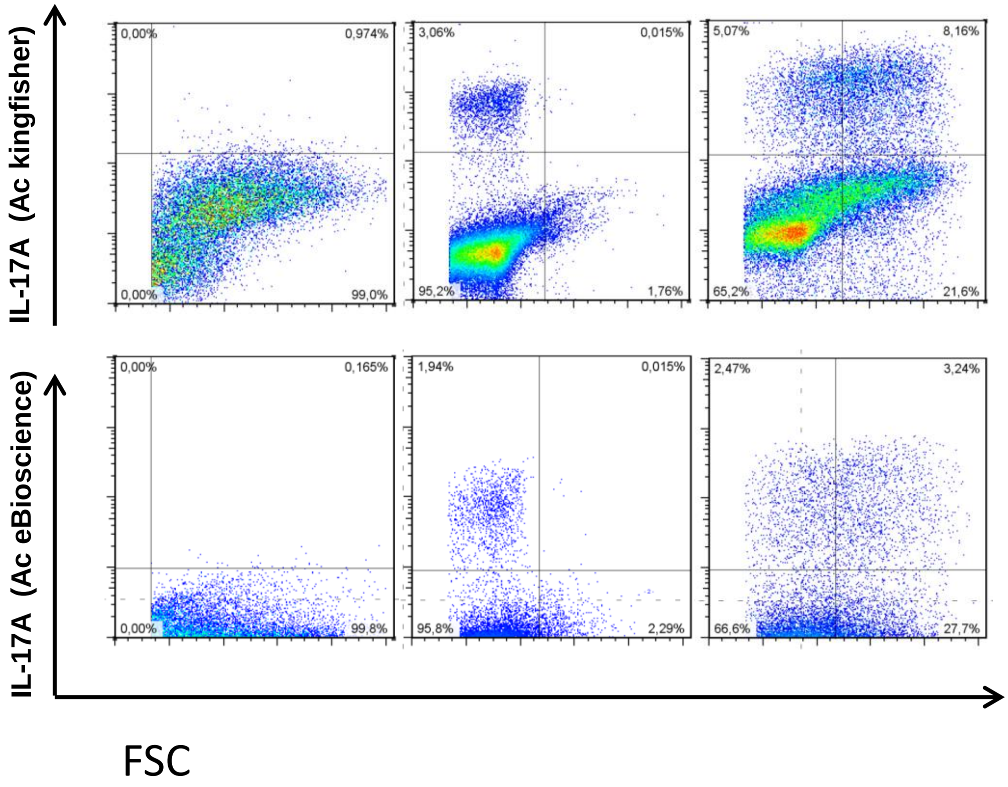

Suppl. Fig. 3. Intracellular labeling with antibodies to IL-17A.

PBMC were isolated from the blood of an immunized cow, restimulated in vitro with ovalbumin for 3 days or left unstimulated, rested for 2 days and finally stimulated with PMA/ionomycin for 5 h with Brefeldin A for the last 3 hours. Cells were then labeled to reveal intracellular IL-17A with either rabbit antiserum to bovine IL-17A (Kingfisher Biotech) followed by RPE-conjugated anti-rabbit antibody, or PE-conjugated mouse monoclonal antibody to human IL-17A (eBioscience). A) isotype control; B) unstimulated cells; C) ovalbumin-stimulated cells. Viable cells are shown.
